# Radiation-Induced Lymphopenia Beyond the COVID-19 Pandemic

**DOI:** 10.3389/fonc.2020.617302

**Published:** 2020-11-18

**Authors:** Giuseppe Carlo Iorio, Umberto Ricardi, Alan Dal Pra

**Affiliations:** ^1^ Department of Oncology, Radiation Oncology, University of Turin, Turin, Italy; ^2^ Department of Radiation Oncology, University of Miami Miller School of Medicine, Miami, FL, United States

**Keywords:** COVID-19, lymphopenia, cancer, radiotherapy, toxicity

The novel human Coronavirus (SARS-CoV-2) pandemic started in late 2019 has killed more than 1 million people worldwide (1). The fatality rate has been linked with a hyperinflammation state dependent of human interleukin-6 levels and a cytokine-release syndrome (2). This pro-inflammatory micro-environment can induce lymphocyte-deficiency *via* apoptosis, impairing immune-homeostasis, and inflammatory-response (3). Indeed, lymphopenia has emerged as a major predictor of severe COVID-19 (3). A lymphocyte count <1.5 × 10^9^/L has been associated with a three-fold increased risk of severe COVID-19 (4). Noteworthy, lymphocytes express Angiotensin-Converting Enzyme 2 (ACE2) receptor which is exploited by the SARS-CoV-2 to enter host target-cells, thus representing a possible virus direct target with a subsequent, potentially lethal, lymphatic organs attack (3).

Cancer treatments can often suppress reservoir lymphoid organs causing lymphopenia, which is associated with an increased risk of opportunistic infections and worse oncologic outcomes due to the lymphocytes essential role within the anti-tumor immune response (5). Although chemotherapy (CT) is often the main trigger of hematologic toxicity (HT), radiotherapy is also a contributing factor for impairment of hematologic cell lines (6, 7). Lymphocytes are extremely radiosensitive and show exponential decline early and throughout irradiation (8). The lethal dose required to reduce the surviving fraction of lymphocytes by 50% (LD50) is only 1.5 Gy, and by 90% (LD90), 3 Gy (9). Radiation-induced lymphopenia (RIL) has been reported to negatively affect prognosis in several neoplasms including non-small cell lung cancer (NSCLC), glioblastoma, pancreatic cancer, esophageal cancer, and head and neck cancers (10–19). Lee et al. evaluated RIL in a cohort of 497 locally advanced pancreatic cancer patients treated with chemoradiation (10). Large radiotherapy volumes and low baseline lymphocyte count predicted for acute severe lymphopenia development and recovery. The authors highlighted the importance of severe acute lymphopenia as it was associated with both poorer overall survival (OS) and progression-free survival (PFS) (10). For NSCLC, Jin et al. considered the immune system as an organ-at-risk in the radiotherapy planning. The *estimated dose of radiation to circulating immune cells* (EDRIC) model was developed, and a higher EDRIC was associated with not only a greater risk of grade 3 or worse lymphopenia but also with poorer oncological outcomes (tumor progression and cancer deaths) within the RTOG-0617 study (12, 13). Tang et al. reported a significant association between lung low-dose exposure (V1-V5), involving the pulmonary vasculature circulating lymphocytes, and lymphopenia degree (14). For esophageal cancers, So et al. found that a low lymphocyte nadir was predictor for OS (hazard ratio = 0.63; p < 0.001) (16). The authors highlighted a significant correlation between radiation dose to circulating immune cells and the lymphocytes nadir (16). Lymphopenia has been previously correlated with vertebral bone marrow (BM) radiation volumes in esophageal cancers (17).

This is in line with data reported for patients undergoing pelvic nodal radiotherapy (PNRT), as BM dose-volume parameters have been associated with the onset of HT, particularly leukopenia (6, 7, 20). Although BM represents only part of the pelvic reservoir lymphoid organs together with lymph nodes, circulating lymphocytes, and gut mucosa, radiation-induced BM suppression is a predictor of leukopenia and lymphopenia (6, 7, 20, 21). BM stem cells are exquisitely radiosensitive and, in the average adult population, half of the hematopoietically active BM (aBM) is located within pelvic bones and lumbar vertebrae (6, 7, 20, 21).

Regarding dosimetric parameters predictive of HT, data from cervical cancer suggest that volumes receiving low-dose are important, e.g., pelvic BM V10 < 90%–95% and V20 < 76%–80% to avoid grade 2-3 HT, especially leukopenia (6, 22). In the RTOG-0418, which included cervical and endometrial cancer patients, BM V40 and median BM dose were linked with higher rates of grade 2 or worse HT (23). Techniques to spare BM, like the employment of PET-guided aBM sparing, have resulted in a significant decrease in HT, particularly grade 3 or worse neutropenia (24).

Similar to cervical cancer, an association between larger volumes receiving low-doses and HT emerged from anal cancer studies (25, 26). Bazan et al. also highlighted the role of the mean pelvic BM dose as a useful toxicity predictor for anal cancer patients, recommending a mean dose at <22.5–25 Gy (25), and supporting the hypothesis of considering the pelvic BM as a parallel organ when predicting HT (25, 27).

Several studies addressed radiation-induced BM suppression in rectal cancer patients undergoing PNRT. In a series of 120 rectal cancer patients treated with neoadjuvant pelvic radiotherapy and concurrent 5-fluorouracil–based CT, Yang et al. found that coxal BM V45 (p = 0.03) and sacral BM V45 (p = 0.03) were associated with a lower leukocyte count and lower absolute neutrophil count ratio at nadir, respectively. Of interest, among all HTs, lymphopenia occurred most frequently: grade 2 lymphopenia was observed in 97.5% of patients while grade 3 in 56.7% (8).

In a mixed population of different pelvic cancers, including rectal, cervical, anal, vaginal, and bladder cancer patients, McGuire et al. reported lymphopenia as the most common recorded toxicity (28).

Chronically lower lymphocytes counts (at 1–2 years after treatment) have been observed in prostate cancer patients undergoing PNRT (20, 29). In this regard, Sini et al. highlighted a higher risk of both acute and late lymphopenia with BM medium-high doses exposure (≥40Gy) (20).

Accordingly, data of an Italian multicentric study with 406 prostate cancer patients treated with PNRT showed an association between large nodal volumes (>900 cc) and acute grade 3 or worse and late grade 2 or worse lymphopenia (lasting at least 1 year) (29). Thus, prostate cancer radiotherapy is a particularly interesting scenario for radiation-induced HT studies, given the increasing use of larger RT volumes to the pelvis.

In times of pandemic, radiation oncologists should be cautious about utilizing large elective radiation volumes, particularly when the benefit is uncertain, to avoid suppression of reservoir lymphoid organs (30). Perhaps short-fractionation schedules, widely preconized during this outbreak (30), could have less impact on hematologic counts, particularly extreme-hypofractionation with steep dose-gradients (11). The low entrance-dose and sharp fall-off of proton therapy have been shown to decrease the RIL risk and severity by minimizing unintended irradiation to circulating immune cells (31). Proton therapy offers dosimetric advantages in BM sparing when delivering PNRT as compared to intensity-modulated radiotherapy and volumetric arc therapy (32, 33). Therefore, stereotactic body radiotherapy (SBRT) and proton therapy may represent promising approaches for “lymphocyte sparing radiotherapy” (21, 31). Additionally, brachytherapy, given the favorable dose distribution to normal tissues, should also be kept in mind for more effective “immune-sparing” radiotherapy approaches (34–37).

Finally, although there is important evidence addressing radiation-induced HT and lymphopenia, there is still a great deal of uncertainty on its long-term oncological implications. In light of the importance of a normal lymphocyte count during the COVID-19 pandemic, we face an unprecedented opportunity for further elucidation of the impact of RIL and “lymphocyte-sparing” strategies.

## Author Contributions

All authors listed have made a substantial, direct, and intellectual contribution to the work, and approved it for publication.

## Conflict of Interest

The authors declare that the research was conducted in the absence of any commercial or financial relationships that could be construed as a potential conflict of interest.
